# Effectiveness of 3 COVID-19 Vaccines in Preventing SARS-CoV-2 Infections, January–May 2021, Aragon, Spain

**DOI:** 10.3201/eid2803.212027

**Published:** 2022-03

**Authors:** Alicia del Cura-Bilbao, Héctor López-Mendoza, Armando Chaure-Pardos, Alberto Vergara-Ugarriza, Joaquín Guimbao-Bescós

**Affiliations:** Miguel Servet University Hospital, Zaragoza, Spain (A. del Cura-Bilbao);; Aragon Department of Health, Zaragoza (A. del Cura-Bilbao, H. López-Mendoza, A. Chaure-Pardos, A. Vergara-Ugarriza, J. Guimbao-Bescós);; University of Zaragoza CASSETEM Research Group, Zaragoza (H. López-Mendoza);; Lozano Blesa University Hospital, Zaragoza (H. López-Mendoza, A. Chaure-Pardos);; GRISSA Research Group, Zaragoza (A. Chaure-Pardos);; Aragon Health Research Institute Foundation (IIS Aragon), Zaragoza (A. Chaure-Pardos)

**Keywords:** COVID-19, vaccine effectiveness, coronavirus disease, SARS-CoV-2, severe acute respiratory syndrome coronavirus disease 2, COVID-19 vaccines, vaccination, respiratory infections, treatment outcomes, Spain, viruses, zoonoses

## Abstract

Reducing severe acute respiratory syndrome coronavirus 2 (SARS-CoV-2) transmission is a worldwide challenge; widespread vaccination could be one strategy for control. We conducted a prospective, population-based cohort study of 964,258 residents of Aragon, Spain, during December 2020–May 2021. We used the Cox proportional-hazards model with vaccination status as the exposure condition to estimate the effectiveness of 3 coronavirus disease vaccines in preventing SARS-CoV-2 infection. Pfizer-BioNTech had 20.8% (95% CI 11.6%–29.0%) vaccine effectiveness (VE) against infection after 1 dose and 70.0% (95% CI 65.3%–74.1%) after 2 doses, Moderna had 52.8% (95% CI 30.7%–67.8%) VE after 1 dose and 70.3% (95% CI 52.2%–81.5%) after 2 doses, and Oxford-AstraZeneca had 40.3% (95% CI 31.8%–47.7%) VE after 1 dose. All estimates were lower than those from previous studies. Results imply that, although high vaccination coverage remains critical to protect people from disease, it will be difficult to effectively minimize transmission opportunities.

Since the beginning of the coronavirus disease (COVID-19) pandemic, one of the main challenges countries have experienced is finding effective ways to reduce illness and death from the disease. Nonpharmaceutical measures have been used extensively, and vaccines were added to the resources of the European Union beginning in December 2020. Results from phase 3 and phase 4 studies have found the vaccines to be highly effective (*1*–*12*). Studies assessing the effectiveness of vaccines in real-world settings among elderly populations (*13*,*14*) have also shown a high effectiveness from a single dose.

Spain has had one of the world’s highest rates of illness and death from COVID-19 (*15*). The Aragon region, in the northeast of the country, has one of Spain’s largest elderly populations; 22% of people among a total population of 1.3 million people are >65 years of age (*16*). Through May 31, 2021, the region had reported 125,465 COVID-19 cases 3,522 deaths (*17*), and a fatality rate of 2.8%. Vaccination programs have proven to be the most effective measure to control the pandemic (*18*) and have been used in conjunction with hygiene and social distancing measures.

The European Union vaccination program started on December 27, 2020. Pfizer-BioNTech (BNT162b2; https://www.pfizer.com), Moderna (mRNA-1273; https://www.modernatx.com), Oxford-AstraZeneca (hAdOx1-S-AZD1222; https://www.astrazeneca.com), and Janssen (https://www.janssen.com) COVID-19 vaccines are currently authorized by the European Medicines Agency (EMA; https://www.ema.europa.eu) for administration in the European Union (*19*). The Pfizer-BioNTech, Moderna, and Oxford-AstraZeneca vaccines have been widely used in Spain and Aragon in accord with the vaccination strategy (*20*,*21*). The Janssen vaccine was added to the vaccination plan later. As of May 31, 2021, 44% of the population of Aragon >18 years of age had been vaccinated with >1 dose of vaccine, and 24.5% had been fully vaccinated (*22*).

The context of coexisting vaccinated and unvaccinated persons and periods of high infection rates among the general population lends urgency to performing vaccine effectiveness (VE) studies. We carried out a cohort study to estimate the effectiveness of vaccination in preventing severe acute respiratory syndrome coronavirus 2 (SARS-CoV-2) infection, in which we compared the Pfizer-BioNTech, Moderna, and Oxford-AstraZeneca COVID-19 vaccines.

## Institutional Review Board Statement

The authors declare that they have complied with the provisions of Spanish Organic Law 3/2018 of December 5 on Personal Data Protection and Digital Rights Guarantee and with the provisions of Regulation (EU) 2016/679 of the European Parliament and of the Council of 27 April 2016 on the protection of natural persons with regard to the processing of personal data and on the free movement of such data, and repealing Directive 95/46/EC (General Data Protection Regulation). Approval for this research was obtained from the Aragon Research Ethics Committee (no. 2021/141).

## Methods

We conducted a prospective, population-based cohort study of residents in the region of Aragon, Spain. Participants were all of the users of the Aragon Health Service, >16 years old of age, who had no evidence of previous SARS-CoV-2 infection, confirmed by reverse transcription PCR (RT-PCR), antigen test, or immunoglobulin G test for SARS-CoV-2 infection at any time before December 27, 2020. We included all residents registered in the Aragon Healthcare System Users Registry (AHSUR) who met the eligibility criteria as of December 31, 2020. AHSUR consists of periodically updated basic demographic data from users of the Aragon Healthcare Service, the public healthcare provider in Aragon. AHSUR contains data from 89% of Aragon inhabitants. We based the study on data collected during December 28, 2020–May 31, 2021.

### Vaccination Program

The goal of COVID-19 vaccination strategy in Spain and Aragon (*20*,*21*) was to protect vulnerable and exposed populations and to achieve full vaccination in as much of the population as possible. Some priority groups were targeted for earlier vaccination during December 2020–February 2021: residents of care (nursing) or residential homes for elderly or disabled people, frontline healthcare workers, caregivers and residential home workers, second-line healthcare workers, and disabled persons not residing in a nursing or residential home. In Aragon, from February 2021 the rollout was expanded to all adults >80 years of age and essential workers—civil protection staff, firefighters, security forces, and educational center staff. In April 2021, the rollout was extended to all adults 60–79 years of age; persons with high-risk conditions and younger age groups have been progressively incorporated into the rollout schedule (*23*).

Specific vaccines were incorporated into the vaccination plan at different times. In Aragon, the Pfizer-BioNTech vaccine was administered beginning December 27, 2020, the Moderna vaccine beginning January 13, 2021, and the Oxford-AstraZeneca vaccine beginning February 7, 2021 (*21*). Because of the stoppage in Oxford-AstraZeneca vaccination in people <60 years of age, those participants receiving that vaccine who we tracked in follow-up had received only 1 dose at the time of the analysis. Because only 8,727 doses of the single-dose Janssen vaccine had been administered since its initiation on April 21, 2021 (*21*), we excluded data on that vaccine from the analysis.

### Exposure Definition (Vaccination Status)

The exposure condition was vaccination status. On each exposure condition, we followed participants, grouped by vaccination status, until that status changed because of SARS-CoV-2 infection, death, loss to follow-up, or end of the study period, whichever occurred first. For first dose vaccination, participants were defined as exposed from 12 days after 1 dose of the Pfizer-BioNTech vaccine, 14 days after 1 dose of the Moderna vaccine, and 21 days after 1 dose of the Oxford-AstraZeneca vaccine according to previous studies. For second dose vaccination, we defined participants as exposed beginning 7 days after 2 doses of the Pfizer-BioNTech vaccine, and 14 days after 2 doses of the Moderna vaccine (*1*–*3*). We defined unvaccinated participants as unexposed.

### Outcome Definition

We considered a participant to be SARS-CoV-2 infected if confirmed by RT-PCR or rapid antigen detection test according to World Health Organization definitions (*24*). Following COVID-19 detection and surveillance guidelines in Spain and Aragon (*25*,*26*), criteria to test for SARS-CoV-2 were having symptoms compatible with COVID-19 or close contact with a person with a laboratory-confirmed SARS-CoV-2 infection diagnosis. We extracted vaccination registry and laboratory testing data from the electronic medical record system of health-related information. The electronic medical record system was automatically updated with those data.

### Patient Characteristics and Confounders

We studied cohort population characteristics to determine if they could potentially act as confounders. These characteristics included age, sex, work or residence in nursing or residential homes, weekly cumulative incidence (WCI) of SARS-CoV-2 infection in each primary care service area, and number of SARS-CoV-2 tests administered in the previous 6 months. We defined SARS-CoV-2 infection WCI as the total number of newly confirmed SARS-CoV-2 infections per 100,000 inhabitants in each primary care service area within the previous 7 days. We extracted data on age, sex, and the primary care service areas from AHSUR. We extracted specific information on nursing and residential homes residents and workers from the Aragon nursing and residential homes information system, an information system to manage care, prevention, and control measures for residents and workers at nursing and residential homes in the context of the COVID-19 pandemic. We used formal tests to compare data between participants lost to follow-up and the studied cohort: χ^2^ tests for all the variables except follow-up time, for which we used Student t-tests, resulting in statistically significant (p<0.01) differences for all the variables.

### Statistical Analysis

We defined the incidence rate (IR) of SARS-CoV-2 infection as the number of confirmed SARS-CoV-2 infections divided by the sum of exposure times for each participant. We computed unadjusted estimators using a Cox proportional-hazards model in which only vaccination status was included, and unadjusted VE against SARS-CoV-2 infection as 1 – hazard ratio. We computed adjusted estimators using a Cox proportional-hazards model and included baseline data on age, sex, and being a resident or worker in a nursing or residential home as categorical covariates in the models. We included WCI from each primary care service area and the number of SARS-CoV-2 tests administered in the previous 6 months as time-variable terms. To introduce the time-variable terms, we split individual follow-up times into weekly intervals. Therefore, we assigned each interval the immediately previous week’s WCI and introduced all intervals into the model as individual observations. We split age and WCI into 4 categories based on percentiles 0–10, 11–50, 51–90, and 91–100. We calculated adjusted VE against SARS-CoV-2 infection as 1 – hazard ratio.

## Results

**Figure 1:**
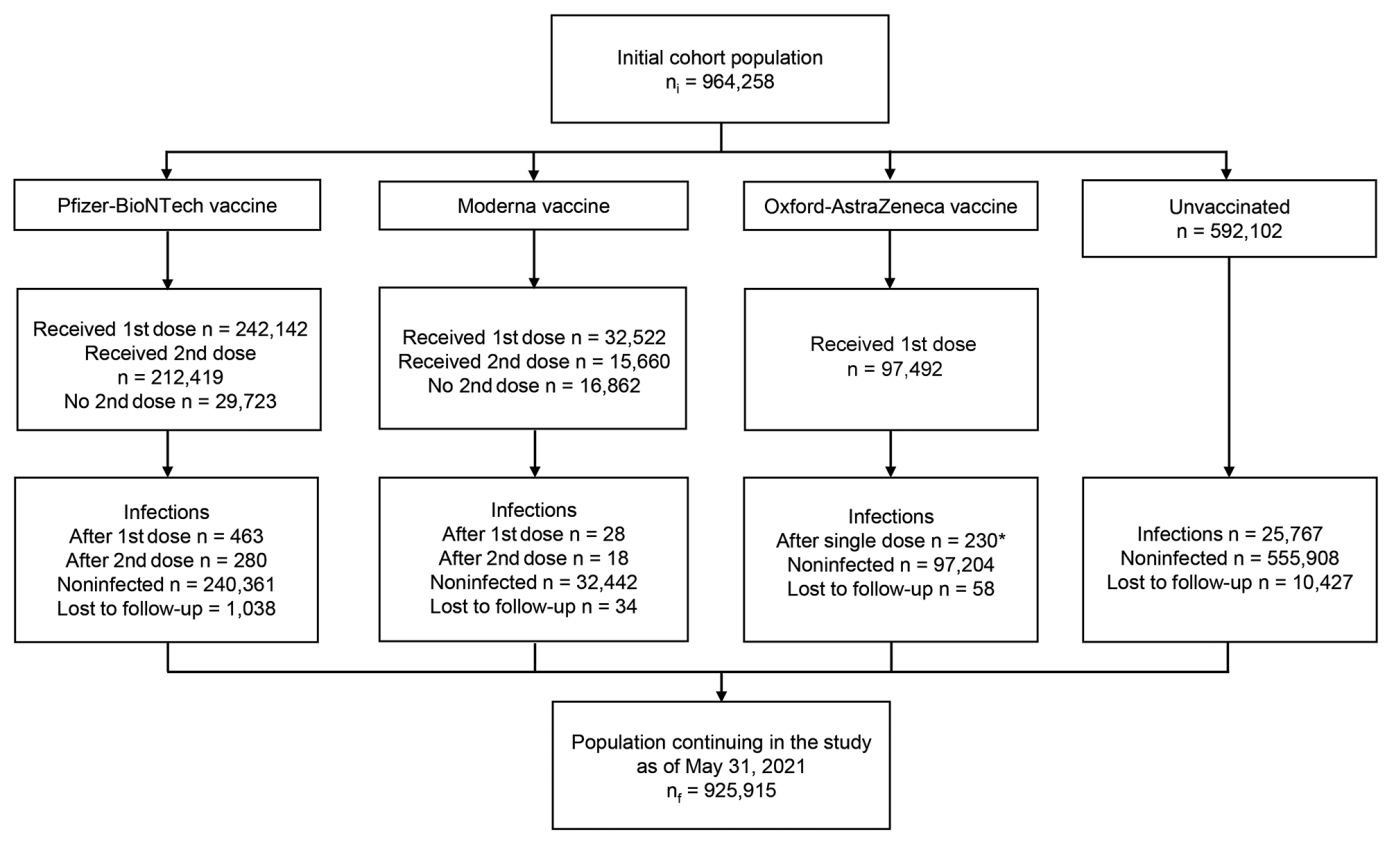
Flowchart of cohort evolution for study of coronavirus disease vaccines in preventing confirmed severe acute respiratory syndrome coronavirus 2 infection, Aragon, Spain, January–May 2021. *Participants vaccinated with the AZ vaccine had all received only 1 dose as of May 31, 2021.

We prospectively followed a cohort of 964,258 people >16 years of age from the general population, corresponding to 72.5% of the population of Aragon; the size and exposure status of the cohort evolved across the study period (Figure 1). We stratified participants’ vaccination exposure by their demographic characteristics (Table 1). As of May 31, 2021, among the participants, 242,142 had been vaccinated with >1 dose of the Pfizer-BioNTech vaccine and 212,419 with 2 doses; 32,522 participants had been vaccinated with at least 1 dose of the Moderna vaccine and 15,660 of them with 2 doses; and 97,492 participants had been vaccinated with 1 dose of the Oxford-AstraZeneca vaccine; 592,102 participants had not yet been vaccinated. We observed differences in the number of Pfizer-BioNTech, Moderna, and Oxford-AstraZeneca vaccines doses administered over the study period, which occurred because of different EMA approval times, vaccine doses available over time, and prioritizing of groups considered for earlier vaccination, specifically persons >75 years old and residents and workers in nursing and residential homes (Table 1). Over the study period, 11,557 (1.2%) participants dropped out of the study; we recorded lost participants by demographic characteristics and causes of withdrawal (Tables 1, 2).

**Table 1:** Characteristics of participants according to vaccination status at endpoint, Aragon, Spain, January–May 2021*

Characteristic	Initial cohort population	PBNT 1st dose		PBNT 2nd dose	MOD 1st dose	MOD 2nd dose		AZ single dose	Unvaccinated		Lost to follow-up
Age group, y											
<25	92,287 (9.6)	1,745 (0.7)		1,489 (0.7)	338 (1.0)	155 (1.0)		3,440 (3.5)	86,764 (14.7)		1,392 (12.0)
25–49	372,525 (38.6)	19,702 (8.1)		16,957 (8.0)	4,230 (13.0)	2,509 (16.0)		19,079 (19.6)	329,514 (55.7)		3,942 (34.1)
50–74	364,754 (37.8)	110,824 (45.8)		86,764 (40.8)	15,541 (47.8)	2,480 (15.8)		74,939 (76.9)	163,45 (27.6)		2,497 (21.6)
≥75	134,692 (14.0)	109,871 (45.4)		107,209 (50.5)	12,413 (38.2)	10,516 (67.2)		34 (0.0)	12,374 (2.1)		3,726 (32.2)
Sex											
F	485,237 (50.3)	143,950 (59.4)		128,280 (60.4)	18,277 (56.2)	10,212 (65.2)		54,132 (55.5)	268,878 (45.4)		5,986 (51.8)
M	479,021 (49.7)	98,192 (40.6)		84,139 (39.6)	14,245 (43.8)	5,448 (34.8)		43,360 (44.5)	323,224 (54.6)		5,571 (48.2)
Site											
Rural	354,418 (36.8)	93,723 (38.7)		82,281 (38.7)	5,154 (15.8)	1,373 (8.8)		35,387 (36.3)	220,154 (37.2)		4,741 (41.0)
Urban	609,840 (63.2)	148,419 (61.3)		130,138 (61.3)	27,368 (84.2)	14,287 (91.2)		62,105 (63.7)	371,948 (62.8)		6,816 (59.0)
Nursing and residential homes											
Residents	11,447 (1.2)	10,847 (4.5)		10,431 (4.9)	11 (0.0)	10 (0.1)		7 (0.0)	582 (0.1)		507 (4.4)
Workers	10,174 (1.1)	8,734 (3.6)		8,570 (4.0)	46 (0.1)	6 (0.0)		155 (0.2)	1,239 (0.2)		33 (0.3)
Follow-up, mean d (SD)	133 (34.9)	15.5 (5.1)		41 (35.3)	19.5 (10.4)	37.9 (23.7)		30.1 (21.7)	148.1 (25.2)		60.1 (33.1)
Total	964,258 (100)	242,142 (100)		212,419 (100)	32,522 (100)	15,660 (100)		97,492 (100)	592,102 (100)		11,557 (100)

*Values are no. (%) participants except as indicated. AZ, Oxford-Astra-Zeneca; MOD, Moderna; PBNT, Pfizer-BioNTech.

**Table 2:** Causes of loss to follow-up during the study period, Aragon, Spain, January–May 2021

Causes	No. patients
Expiration of service*	3,328
Death	2,903
Change of residence to another region of Spain	2,020
Loss of entitlement†	250
Change of residence to another country	15
Duplicate user‡	2
Unknown	3,039

*Aragon Health Service healthcare ended for administrative reasons. Most common were expiration of temporary service for persons who moved from another self-governing region of Spain for a specific period of time (maximum 6 months), subject to renewal; and for foreign citizens with no residence license who had not applied for renewal of Aragon Health Service–provided healthcare in 2 years. †Loss of entitlement to Aragon Health Service–provided healthcare when person begins working unless they renounce mutual insurance company–provided healthcare (applies to a few public workers in Spain whose healthcare provider is a mutual insurance company). ‡Health record of participant was duplicated in the Aragon Healthcare System Users Registry.

The 592,102 unvaccinated participants had 25,767 SARS-CoV-2 infections and an IR of 1.41/1,000 person-weeks. The 242,142 participants vaccinated with 1 dose of the Pfizer-BioNTech vaccine had 463 infections (IR 0.86) and the 212,419 with 2 doses had 280 infections (IR 0.23). The 32,522 participants vaccinated with 1 dose of the Moderna vaccine had 28 infections (IR 0.31) and the 15,660 with 2 doses had 18 infections (IR 0.21). The 97,492 participants vaccinated with 1 dose of the Oxford-AstraZeneca vaccine had 230 infections (IR 0.55).

### Unadjusted Vaccine Effectiveness against SARS-CoV-2 Infection

The Pfizer-BioNTech vaccine had 23.5% (95% CI 16.0%–30.3%) unadjusted VE against SARS-CoV-2 infection after 1 dose and 76.1% (95% CI 73.1%–78.8%) after 2 doses. The Moderna vaccine had 69.2% (95% CI 55.4%–78.8%) unadjusted VE after 1 dose and 78.4% (95% CI 65.6%–86.4%) after 2 doses. The Oxford-AstraZeneca vaccine had 43.7% (95% CI 35.7%–50.7%) unadjusted VE after 1 dose (Table 3).

**Table 3:** Effectiveness of Pfizer-BioNTech, Moderna, and Oxford-AstraZeneca coronavirus disease vaccines in preventing confirmed SARS-CoV-2 infection, Aragon, Spain, January–May 2021*

Vaccination status	Person-days, total (average)	Population	SARS-CoV-2 infections	IR†	Unadj HR‡	Adj HR‡	Unadj VE,§ % (95% CI)	Adj VE, § % (95% CI)
Pfizer-BioNTech								
1 dose	3,750,582 (15.5)	242,142	463	0.86	0.77	0.79	23.5 (16.0–30.3)	20.8 (11.6–29.0)
2 doses	8,705,040 (41.0)	212,419	280	0.23	0.24	0.30	76.1 (73.1–78.8)	70.0 (65.3–74.1)
Moderna								
1 dose	633,821 (19.5)	32,522	28	0.31	0.31	0.47	69.2 (55.4–78.8)	52.8 (30.7–67.8)
2 doses	592,877 (37.9)	15,660	18	0.21	0.22	0.30	78.4 (65.6–86.4)	70.3 (52.2–81.5)
Oxford-AstraZeneca								
1 dose	2,932,610 (30.1)	97,492	230	0.55	0.56	0.60	43.7 (35.7–50.7)	40.3 (31.8–47.7)
Unvaccinated	128,261,888 (133.0)	592,102	25,767	1.41	1.00	1.00	NA	NA

*Adj, adjusted; HR, hazard ratio; IR, incidence rate; NA, not applicable; SARS-CoV-2, severe acute respiratory syndrome coronavirus; unadj, unadjusted; VE, vaccine effectiveness †Incidence rate of SARS-CoV-2 infection was measured in 1,000 person-weeks (not person-days) to make it to read the table (estimates expressed with <2 decimals. ‡HR was adjusted by age, sex, work or residence in nusing or residential homes), weekly cumulative incidence in each primary care service area, and number of SARS-CoV-2 tests administered in the previous 6 months. §Vaccine effectiveness against SARS-CoV-2 infection was calculated as 1 – HR.

### Adjusted Vaccine Effectiveness against SARS-CoV-2 Infection

After adjusting for age, sex, work or residence in a nursing or residential home, WCI in each primary care service area, and number of SARS-CoV-2 tests administered in the previous 6 months we found that the Pfizer-BioNTech vaccine had 20.8% (95% CI 11.6%–29.0%) adjusted VE after 1 dose and 70.0% (95% CI 65.3%–74.1%) after 2 doses. The Moderna vaccine had 52.8% (95% CI 30.7%–67.8%) adjusted VE after 1 dose and 70.3% (95% CI 52.2%–81.5%) after 2 doses; and the Oxford-AstraZeneca vaccine had 40.3% (95% CI 31.8%–47.7%) adjusted VE after 1 dose (Table 3).

### SARS-CoV-2 Infection Cumulative Risk Curves

**Figure 2:**
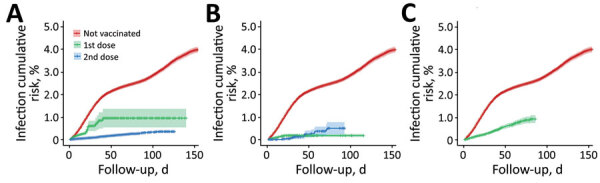
Cumulative risk curves (1 minus the Kaplan-Meier risk) of severe acute respiratory syndrome coronavirus 2 (SARS-CoV-2) infection for 3 coronavirus disease vaccines, Aragon, Spain, January–May 2021. A) BioNTech-Pfizer BNT162b2 mRNA, B) Moderna mRNA-1273, and C) Oxford-AstraZeneca ChAdOx1-S-AZD1222. Shadows across lines represent 95% CI. For unvaccinated participants, 95% CI at day 90 of follow-up was 2.6%–2.8%. For participants who went on to receive the BioNTech-Pfizer vaccine, 95% CI at day 90 of follow-up was 0.5%–1.4% (1 dose) and 0.3%–0.4% (2 doses). For the Moderna vaccine, 95% CI at day 90 of follow-up was 0.1%–0.2% (1 dose), and 0.2%–0.8% (2 doses). For Oxford-AstraZeneca, 95% CI at day 90 of follow-up was 0.7%–1.0% (1 dose). Cumulative risk curves of SARS-CoV-2 infection start from the day after vaccination when full protection against SARS-CoV-2 infection is thought to begin, according to previous studies (*1*–*3*). The hairs on both sides of the lines represent participants lost to follow-up; gaps represent periods of time between losses.

For unvaccinated participants, the risk for SARS-CoV-2 infection rose to 2% at day 44 and to 4% at day 154 of follow-up. For participants who received 1 dose of the Pfizer-BioNTech vaccine, the risk rose to 1% at day 40 of follow-up, but remained <1% during the entire follow-up period (120 days) for those with 2 doses (Figure 2, panel A). For participants who received 1 dose of the Moderna vaccine, risk remained <0.5% during the entire follow-up time (120 days) and for participants vaccinated with 2 doses, the risk rose from 0% to 0.5% during days 30–71, then remained at 0.5% until the end of follow-up (day 90; Figure 2, panel B). For participants who received 1 dose of the Oxford-AstraZeneca vaccine, risk rose to 0.9% after 80 days of follow-up (Figure 2, panel C).

## Discussion

In the general population, our findings showed an effectiveness of 3 different vaccines against SARS-CoV-2 infection, but with lower efficacy estimates than from clinical trials and other VE studies. We found 20.8% VE after 1 dose of the Pfizer-BioNTech vaccine and 70.0% after 2 doses; for the Moderna vaccine, these numbers were 52.8% VE after 1 dose and 70.3% VE after 2 doses, and for the Oxford-AstraZeneca vaccine, 40.3% after 1 dose.

For the Pfizer-BioNTech and Moderna vaccines, these values were lower than those in other observational studies, which had ranges of 61.9%–80% VE after 1 dose and 90%–96% VE >7 days after 2 doses (*8*,*9*,*27*–*29*). These differences could possibly be explained by the population-wide design of our study, which included a higher percentage of elderly persons in the Pfizer-BioNTech–vaccinated group than in the other studies. In contrast, our results showed a higher VE after 2 doses of the Pfizer-BioNTech vaccine than the 65% VE found in another study (*30*), probably because they used a different approach for estimating VE that included only close contacts of positive cases and assigned every person in the cohort the same observation period and as a result vaccinated and unvaccinated participants most likely experienced similar exposure to SARS-CoV-2.

Our findings indicated a higher VE (52.8%) after 1 dose of the Moderna vaccine than after 1 dose of either the Pfizer-BioNTech or Oxford-AstraZeneca vaccines and similar VEs after 2 doses of both the Moderna and Pfizer-BioNTech vaccines. However, our results did not reach the VE estimates of 83% after 1 dose and 82% after 2 doses of Moderna vaccine found in another study (*28*). The small sample size in that study, which only included healthcare personnel and other essential workers, might explain these differences in VE. However, as in that study (*28*), VE after 1 and 2 doses of the Moderna vaccine were also very close.

Safety concerns resulted in the suspension of the Oxford-AstraZeneca vaccine before anyone in our cohort received a second dose, and therefore we estimated VE only after 1 dose (40.3%), similar to the 44% VE after 1 dose of the Oxford-AstraZeneca vaccine in another article (*30*). In contrast, another study found a VE of 60% against symptomatic disease after a single dose of the Oxford-AstraZeneca vaccine in adults >70 years of age, as expected because of the study’s more severe outcome measures and exclusively elderly population (*14*).

Cumulative risk curves of SARS-CoV-2 infection show that the cumulative risk of infection in unvaccinated participants rose to 4% at day 154 of follow-up whereas the risk remained <1% during the entire follow-up period (120 days) in fully Pfizer-BioNTech–vaccinated participants, results consistent with those from a nationwide study (*8*). Risk remained <0.5% in participants vaccinated with 1 dose of the Moderna vaccine during the entire follow-up time (120 days) and <1% during the entire follow-up time (90 days) in fully vaccinated participants. In the participants with 2 doses of the Moderna vaccine, the slight increase in risk from day 30 onwards might be explained by the relatively small number of participants from our cohort who were vaccinated with the second dose and reached long follow-up times (>50 days), which can cause instability of estimates for prolonged follow-up times. For the Oxford-AstraZeneca vaccine, the difference in risk between unvaccinated participants and those vaccinated with 1 dose (2.5% vs. 0.9% at day 80 of follow-up) highlights the VE after 1 dose of the Oxford-AstraZeneca vaccine.

One limitation of our study was losses to follow-up because of administrative leaves from AHSUR. Participants lost to follow-up were statistically different from the studied cohort. Nevertheless, they represent only 1.2% of the initial population, which limited the magnitude of this bias. Timing of vaccine rollout also varied between priority groups, targeted for earlier vaccination, and the general population. This difference may have affected the results by adding more variability, particularly because Pfizer-BioNTech was mostly used in population ≥75 years of age, who were vaccinated earlier, whereas Oxford-AstraZeneca was mostly used in general population, who were vaccinated at a later time.

Research has documented that the proportion of symptomatic infections in vaccinated persons is lower than in unvaccinated ones because vaccination prevents symptoms (*28*). Therefore, studies based on symptomatic persons (*1*–*7*,*11*,*13*,*14*) underestimate the total infection rate in vaccinated persons to a greater extent than in unvaccinated ones and consequently overestimate VE. Our study included all confirmed symptomatic and asymptomatic SARS-CoV-2 infections, and thus it would be expected that VE would be lower than in studies only including symptomatic disease and our VE estimates more relevant to transmission control, because in real-world conditions, symptomatic and asymptomatic infections coexist and both contribute to transmission.

Similarly, according to COVID-19 detection and surveillance guidelines in Spain and Aragon (*25*,*26*), tests were administered less frequently to asymptomatic than to symptomatic persons, resulting in underdetection of asymptomatic infections. This bias was reduced because underdetection occurred in both vaccinated and unvaccinated persons but could still lead to overestimating VE. On the other hand, also following the detection program guidelines, tests were administered to close contacts regardless of their vaccination status, which reduced the chance of detection bias in our study. However, routine screenings carried out in nursing and residential homes could have altered our findings if there were more screenings in vaccinated than in unvaccinated participants. The role of dominant variants of concern in the transmission was unknown at the time of our data analyses. The rapid circulation of these variants may have introduced confounding, but it was minimized by including weekly variability, and therefore calculated VE estimates represent a summarized measure against all variants, adjusted by incidence. Practical factors such as hygiene and social distance measures might also have affected the estimates of VE.

Our study shows great strength in statistical power because of the large population cohort and use of a refined methodology. Risk of infection differed between participants according not only to vaccination status but also to the evolution of the epidemic curve. For this reason, we used an approach of weekly repeated measures, adjusted by WCI in each primary care service area.

In conclusion, we found effectiveness against SARS-CoV-2 infection for Pfizer-BioNTech, Moderna, and Oxford-AstraZeneca vaccines to be lower than efficacy estimates from clinical trials and other VE studies. Even if high vaccination coverages are reached in the general population (*31*,*32*), effectively minimizing transmission opportunities might be limited, because age groups of persons <12 years of age were not being immunized at the time of our data gathering. Even so, reaching high vaccination coverage is important to decrease SARS-CoV-2 transmission in the general population.
